# A bibliometric analysis and review of recent researches on TRPM7

**DOI:** 10.1080/19336950.2020.1788355

**Published:** 2020-07-09

**Authors:** Shiqi Zhang, Dongyi Zhao, Wanying Jia, Yuting Wang, Hongyue Liang, Lei Liu, Wuyang Wang, Zhiyi Yu, Feng Guo

**Affiliations:** a Department of Pharmaceutical Toxicology, School of Pharmaceutical Science, China Medical University, Shenyang, China; b Human Aging Research Institute, School of Life Sciences, Nanchang University, Nanchang, Jiangxi, China;; c Jiangsu Province Key Laboratory of Anesthesiology, Xuzhou Medical University, Xuzhou, China; d Division of Medicinal Chemistry, School of Pharmaceutical Sciences, Shandong University, Shandong, China

**Keywords:** Bibliometric, TRPM7, CiteSpace V, WoSCC

## Abstract

The transient receptor potential melastatin-subfamily member 7 (TRPM7) is a ubiquitously expressed protein that contains both an ion channel and an active kinase. TRPM7 has involved in a variety of cellular functions and critically participates in various diseases mainly including cancer and neurodegenerative disorders. However, the theme trends and knowledge structures for TRPM7 have not yet been studied bibliometrically. The main purposes of this research are to compare the scientific production in the research field of TRPM7 among countries and to evaluate the publication trend between 2004 and 2019. All publications were extracted from the Web of Science Core Collection (WoSCC) database from 2004 to 2019. Microsoft Excel 2018, Prism 6, and CiteSpace V were applied to analyze the scientific research outputs including journals, countries, territories, institutions, authors, and research hotspots. In this report, a total of 860 publications related to TRPM7 were analyzed. Biophysical Journal ranked top for publishing 31 papers. The United States of America had the largest number of publications (320) with a high citation frequency (11,298) and H-index (58). Chubanov V (38 publications) and Gudermann T (38 citations), who from Ludwig Maximilian University of Munich, were the most productive authors and had the greatest co-citation counts. Our study also combined the bibliometric study with a systematic review on TRPM7, highlighting the four research frontiers of TRPM7. This is the first study that demonstrated the trends and future development in TRPM7 publications, providing a clear and intuitive profile for the contributions in this field.

Transient receptor potential (TRP) proteins belong to a diverse family of ion channels that are present in multiple types of tissues. They function as gatekeepers by mediating ion fluxes in response to various sensory stimulus including temperature, vision, taste, and pain [1]. In mammals, the TRP superfamily includes 28 members, which can be subdivided into 6 subfamilies: TRPA (ankyrin), TRPC (canonical), TRPM (melastatin), TRPML (mucolipin), TRPP (polycystin), and TRPV, but humans only express 27 TRP channel members [2,3]. There are eight members in the TRPM subfamily (TRPM1–8), and each member consists of six transmembrane domains with a pore‐forming loop, and the N‐ and C‐termini directed to the cytoplasm. By mediating fluxes of cations, TRPM channels regulate diversified types of physiological processes such as night vision (TRPM1), immune TRPM3), artery vasoconstriction (TRPM4), taste‐signaling and insulin secretion (TRPM5), magnesium absorption (TRPM6 and TRPM7), and cold sensation (TRPM8) [2,4].

Similar to the other TRPs, TRPM7 comprises tetrameric ion channels with each subunit containing six-transmembrane segments. The TRPM7 channel loses its fourfold symmetry at its intracellular enzymatic domain. This structural feature resembles that of the tetrameric ionotropic glutamate receptors whose extracellular ligand-binding domains form dimers [5,6]. Most TRP channels are poorly selective for conducting cations, while TRPM7 is selective for divalent cations [7,8]. The TRPM7 channel conducts cations, specifically calcium (Ca^2+^), magnesium (Mg^2+^) and zinc (Zn^2+^), and is implicated in cellular and systemic Mg^2+^ homeostasis, Zn^2+^-mediated toxicity and intracellular Ca^2+^ signaling. The activity of TRPM7 channel is considered to be regulated by intracellular cations (Mg^2+^, Ba^2+^, Sr^2+^, Zn^2+^, Mn^2+^), Mg-ATP, polyamines, chloride (Cl^−^) and bromide (Br^−^) ions, pH, and phospholipid phosphatidylinositol 4,5-bisphosphate (PIP2) [9]. Furthermore, TRPM7 channel is involved in a variety of diseases including neurological disorder, cancer, tooth pain, etc.

Over the past 15 years, significant progresses of molecular and functional characterization of TRPM7 have been made in peripheral tissues and cell lines, while the kinase and channel encoding portions of the TRPM7 gene domains are still not under exploration [10].

A bibliometric study is an effective way to calculate the overall trend of the research activity and to clarify the connections between relevant research institutions [11]. Bibliometrics are also able to evaluate the amount and evolution of scientific productions among countries and years in major biomedical fields, and are particularly useful for novel disciplines whose impacts on the larger field of biomedical study have yet to be fully evaluated [12]. However, to date, no bibliometric studies have been published to demonstrate the trends in TRPM7 research activity. In this research, we provided a report on the scientific production in the field of TRPM7 among countries during 16 years (2004 − 2019).

In this study, we applied bibliometrics to dissect the characteristics of scientific articles in Web of Science (Thomson Reuters Company) into several components so that the subjective factors are minimalized, and then analyzed the overall publication trends of TRPM7 channel.

## Data sources and search strategies

Literatures were retrieved online through the Thomas Reuters Web of Science (WoS) bibliographic database on 31 December 2019. To ascertain the trend in publications, we collected 15 years’ worth of data from 2004 to 2019. All literature retrieval and record downloads were completed on 31 December 2019 to avoid changes in citation counts caused by daily database update. The search keywords entered into the database were as follows: TRPM7 AND LANGUAGE: (English) AND DOCUMENT TYPES: (ARTICLE OR PROCEEDINGS PAPER). The data were downloaded directly from the database as secondary data without further animal experiments, and thus, no ethical approval was required.

## Data collection

Initially, the records retrieved from WoS were downloaded, screened, sorted, and extracted. Then, these data were converted to.txt format, and imported into CiteSpace V 5.1.R8 SE, 64bit and the Online Analysis Platform of Literature Metrology for data analysis.

## Statistical analysis

We reviewed the characteristics of publications by establishing “The WoS Literature Analysis Report” online, including distribution of countries/regions, institutions, journals and authors, number of annual publications, citation counts, and H-index. The “H-index” has been indicated as a dependable method of predicting future research, and includes the time-cited publications belonging to a given country compared to the number of times for which those publications are at least co-cited [11]. A comparison of publication quantity, citations, and H-index between different countries was organized by using GraphPad Prism 6. The statistical results were then displayed by using CiteSpace V [13]. The consequence and number of co-cited authors and co-cited references were calculated via VOSviewer (Leiden University, Leiden, Netherlands).

## General information and annual publication output

**Figure 1.**
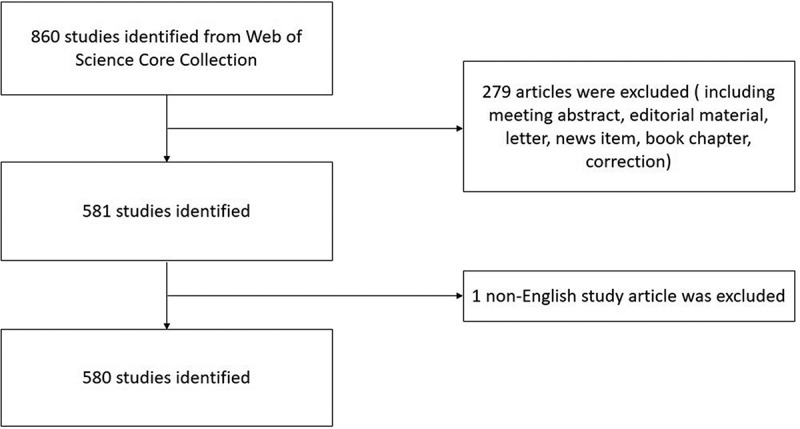
The flowchart of TRPM7 researches.

A total of 860 studies were searched from the WoS database, including 581 articles (67.56%), 165 meeting abstracts (19.19%), and 87 reviews (10.12%) (Table 1). From the collection, only one article was published in French. The search criteria produced 860 pieces of literature, and the flowchart of literature including these terms is shown in Figure 1.

**Table 1.** The type of the retrieved documents.

Rank	Type of documents	Frequency N = 860	Percentage
1	Article	581	67.56%
2	Meeting Abstract	165	19.19%
3	Review	87	10.12%
4	Editorial Material	14	1.63%
5	Proceeding Paper	14	1.63%
6	Book Chapter	6	0.70%
7	Correction	4	0.47%
8	News Item	4	0.47%
9	Letter	3	0.35%

**Figure 2.**
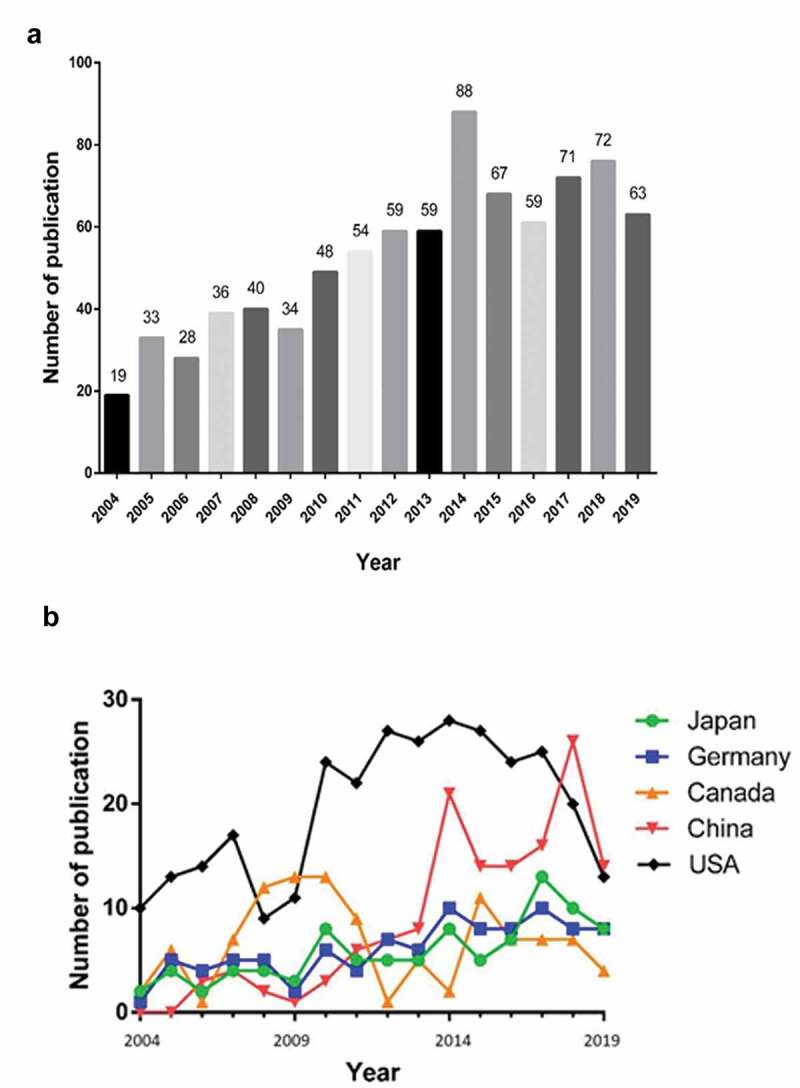
Publication outputs and growth prediction. (a) The number of annual publications on TRPM7 research from 2004 to 2019; and (b) The line chart of publications trend on TRPM7 among different countries.

The number of publications remained steady in general, but decreased slightly after 2014. Moreover, the overall trend increased from 19 papers in 2004 to 63 papers in 2019, and the published articles in 2014 were more than the sum of papers from all other years (Figure 2a). While the trend of worldwide TRPM7 research publications remained stable in these years, several countries had variable publication data outputs. Overall, the publication outputs from the top 5 countries increased continuously between 2004 and 2019, albeit that publications outputs decreased between 2007 and 2009 in these countries except Canada (Figure 2b).

## Citation and H-index analysis

**Figure 3.**
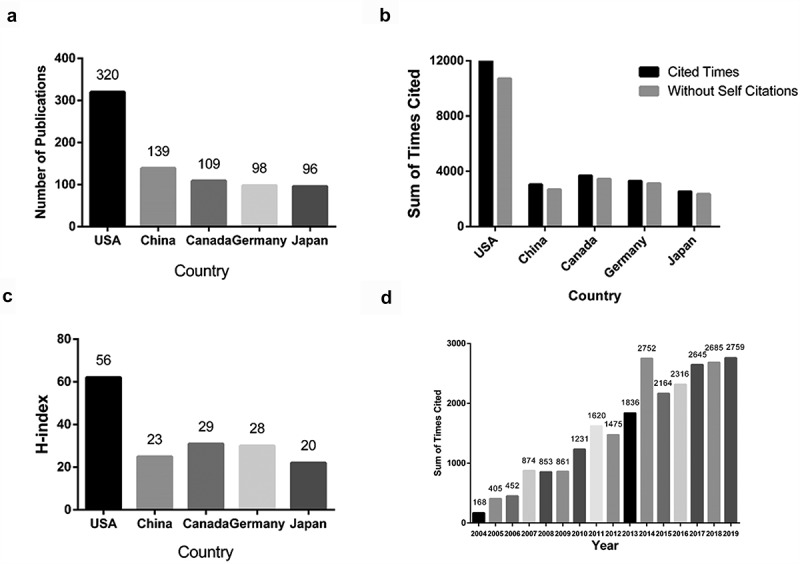
The publications (a), citation counts (b), and H-index (c) in the top 5 countries; and (d) The number of cited times on TRPM7 researches from 2004 to 2019.

Based on our analysis of the WoS database, the USA had published the majority papers during these 15 years, followed by China (Figure 3a). Papers from the USA received the highest number of citations (12,843), accounting for 50.15% of the total number. The H-index of papers from the USA was 56. Canada ranked second with 3,683 citations (14.38%) and H-index of 29. Germany had a citation frequency of 3,291 (12.85%) and a H-index of 28. Notably, China possessed the second largest number of published papers, while these papers had been cited 3045 times and the H-index of China was 23 (Figure 3b and C). All papers related to TRPM7 were cited 25,611 times from 2004 to 2019 (16,991 times excluding the self-citations). The average citation frequency of each paper was 29.78. The number of cited times is shown annually from 2004 to 2019 in Figure 3d. The overall number of cited times in this field remained growing during these 15 years. For instance, the number of cited times was solely 16 in 2004, while it had already reached 2532 in 2018.

**Figure 4.**
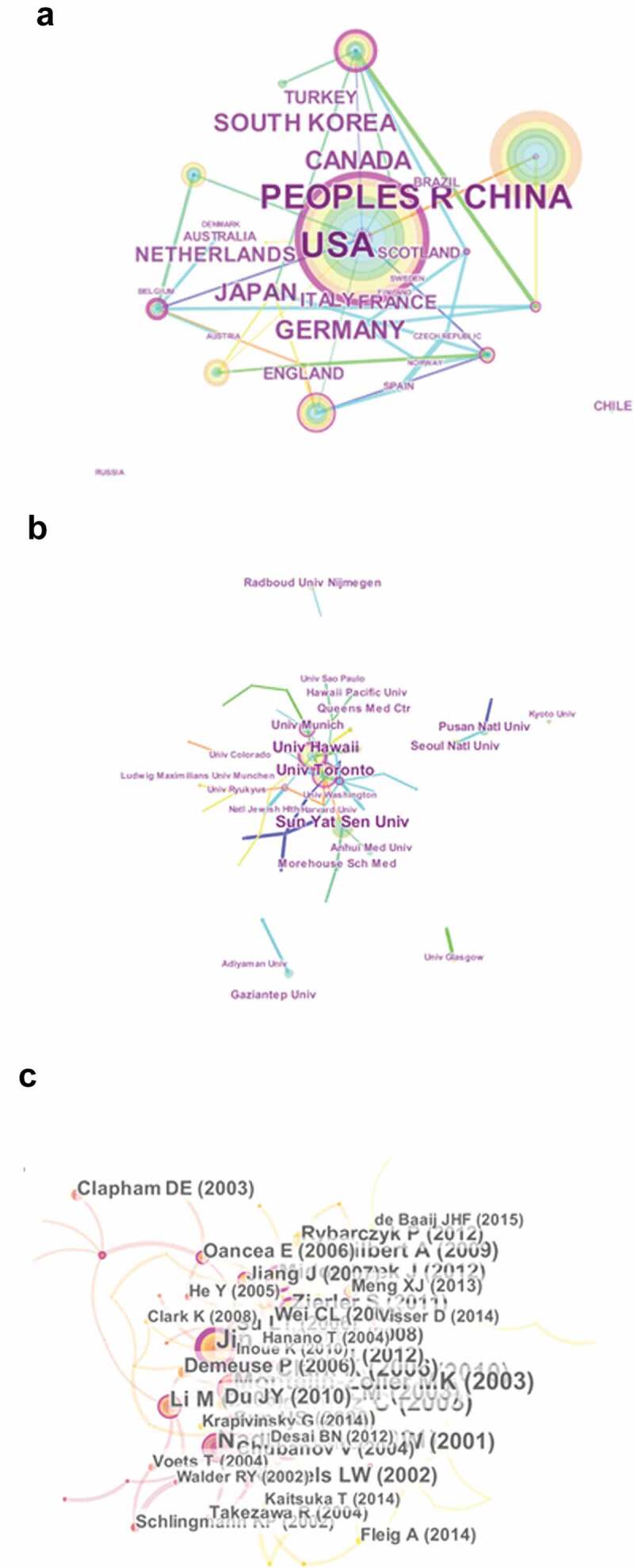
The distribution of countries/regions and institutions. (a) The network map of countries/regions, (b) the network map of institutions, and (c) the network map of authors that conducted TRPM7 researches.

Researchers from 38 countries contributed to the research progress of TRPM7. The USA published the largest portion of papers (320, 37.21%), followed by China (139, 16.16%) and Canada (109, 12.67%). The data contributed by different institutions and countries are shown in Table 2 and Figure 3a, and the connection among these countries is displayed in the network (Figure 4a). Most of the publications were originated from institutions in North America (Figure 4b and C) with University of Toronto producing the highest number of publications on TRPM7 (43), followed by University of Hawaii (33), Seoul National University (28) and Harvard University (27) (Table 2). Around 2,733 authors contributed over 860 articles involving in TRPM7. The networks shown in Figure 6 indicate the cooperation among these authors. Chubanov V and Gudermann T were the most productive with 38 publications on TRPM7, followed by Touyz RM (37 papers) and Fleig A (30 papers) (Table 3).

**Table 2.** The top 8 Institutions contributed to the publications on TRPM7 researches.

Rank	Institution	Frequency N = 860	Percentage
**1**	University of Toronto	43	5.00%
**2**	University of Hawaii	33	3.84%
**3**	Seoul National University	28	3.26%
**4**	Harvard University	27	3.14%
**5**	Sun Yat-sen University	26	3.02%
**6**	University of Medicine and Dentistry of New Jersey	26	3.02%
**7**	Radboud University Nijmegen	23	2.67%
**8**	University of Washington	23	2.67%
**9**	Ludwig-Maximilians-University Munich	20	2.32%
**10**	Pusan National University	20	2.32%

**Table 3.** The Top 10 authors that published articles on TRPM7 researches.

Rank	Author	Frequency N = 860	Percentage
**1**	Chubanov V	38	4.42%
**2**	Gudermann T	38	4.42%
**3**	Touyz RM	37	4.30%
**4**	Fleig A	30	3.49%
**5**	Kim BJ	25	2.90%
**6**	Runnels LW	24	2.79%
**7**	Matsushita M	23	2.67%
**8**	Kozak JA	21	2.44%
**9**	Penner R	21	2.44%
**10**	Schmitz C	21	2.44%

## Active journals

**Figure 5.**
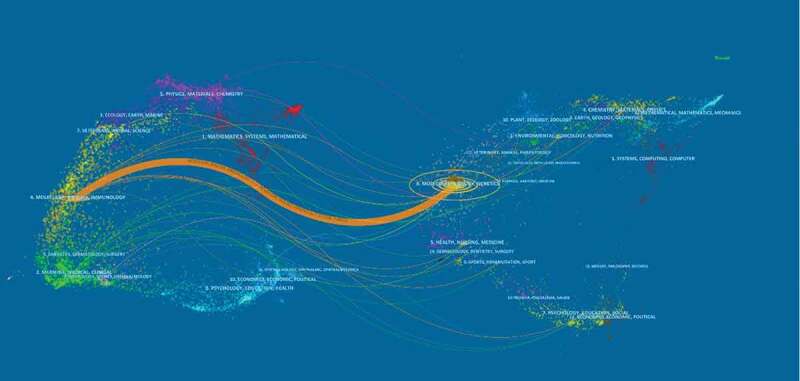
The dual-map overlay of journals related to TRPM7 researches.

**Figure 6.**
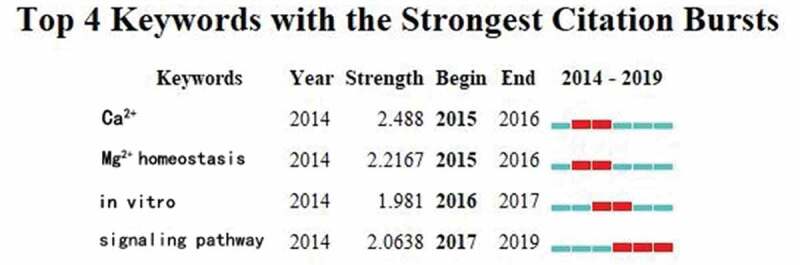
The keywords with the strongest citation bursts of publications on TRPM7 researches.

In total, 274 journals have published TRPM7 papers and 25 journals have published more than six papers in this field, comprising approximately 9.12% of all published literature. The top 10 journals in terms of the number of publications are indicated in Table 3. The Biophysical Journal (IF = 3.665, 2019) had the highest number of 31 (3.60%), while the Journal of Biological Chemistry (IF = 4.106, 2019) and Plos One (IF = 2.776, 2019) published 26 papers (3.02%) on TRPM7. The Scientific Report (IF = 4.011, 2019) ranked the fourth with 22 papers (2.56%). A dual-map overlay of the journals is presented in Figure 6. This map overlay indicates that most papers were published in the fields of mathematics, environmental, ecology, chemistry, physics, and psychology. They mainly cited journals in the areas of molecular research, biology, and genetic (Table 4 and Figure 5).

**Table 4.** The Top 11 journals that published articles on TRPM7 researches.

Rank	Journal Tittle	Country	Frequency N = 860	Percentage	IF2019
**1**	BIOPHYSICAL JOURNAL	United States	31	3.60%	3.665
**2**	JOURNAL OF BIOLOGICAL CHEMISTRY	United States	26	3.02%	4.106
**3**	PLOS ONE	United States	26	3.02%	2.776
**4**	SCIENTIFIC REPORTS	England	22	2.56%	4.011
**5**	NAUNYN-SCHMIEDEBERGS ARCHIVES OF PHARMACOLOGY	Germany	20	2.38%	2.058
**6**	MAGNESIUM RESEARCH	England	17	1.98%	1.588
**7**	CELL CALCIUM	Scotland	16	1.86%	3.932
**8**	PROCEEDINGS OF THE NATIONAL ACADEMY OF SCIENCES OF THE UNITED STATES OF AMERICA	United States	15	1.74%	9.58
**9**	CANCER RESEARCH	United States	15	1.74%	8.378
**10**	HYPERTENSION	United States	14	1.63%	7.017

## Frequently encountered terms

By extracting the TRPM7 relevant publications via CiteSpace V, keywords were identified and analyzed via the strong citation bursts (Figure 6). The keywords that had strong bursts after 2004 were as follows: “Ca^2+^” (2015–2016), “Mg^2+^ homeostasis” (2015–2016), “invitro” (2016–2017), and “signaling pathway” (2017–2019).

We used CiteSpace, a scientific mapping software tool, for a bibliometric analysis of relevant TRPM7 researches as well as a visual description of the research status and trends, which allowed a systematic understanding of the past and future.

## General information

We used a total of 860 publications to calculate a fluctuating growth trend over time. Our data regarding trends in publication years were consistent with two phases. The first phase (2004–2014) can be considered to continue being more popular for TRPM7 researches, while the second phase (2015–2019) tends to be a stable period. Undoubtedly, the USA made predominant contributions to TRPM7 researches, but China, Canada and Japan played a critical role in advancing this field as well.

Institutions with tremendous scientific research strengths were observed to be mainly converged in higher education research institutions according to the publication counts and centrality, and these institutions are important bases for medical scientific research and education. The top 10 institutions contributed to 247 articles, accounting for 32.22% of all publications. In this list, four of them (Harvard University, University of Hawaii, University of Medicine and Dentistry of New Jersey, and University of Washington) were located in the USA, indicating that institutions located in the USA occupied the top rankings in terms of absolute contributions and relative influences, which is consistent with the analysis of contributions of countries in the field of TRPM7. Due to the close relationship between the level of health care and the speed of economic development, the institutional distribution provides invaluable information for researchers to identify and choose appropriate collaborative institutions.

In terms of the top 15 academic journals, eight journals had an impact factor higher than 4. Among these journals, the Proceedings of The National Academy of Sciences of the USA (IF = 9.580, 2019) had the highest H-index of 11, and it occupied 1.74% publications from all journals. The impact factors of Cancer Research (IF = 8.378, 2019), Hypertension (IF = 7.017, 2019), and Circulation Research (IF = 15.860, 2019) surpassed 7 as well. These four journals contained 54 articles, accounting for 6.28% of all publications. Conclusively, articles published in high-impact journals account for one-fifth of the total numbers of publications, and thus, the quality of related researches in the field of TRPM7 is well enough but remains to be further improved.

We found that the USA maintained the dominant position in terms of publications, citation frequency and H-index, suggesting that the USA exerts a crucial role in this field. In contrast, China plays an advantageous role in terms of publication numbers (16.16%, 139), while its citation and H-index are arranged at the fourth position. Encouragingly, China is the only developing country in the top five countries including two North-American countries, two Asian-Pacific countries and one European country. A strong collaboration among countries can stimulate research, and in this case, strong collaborations were found among the USA, China, Canada, Japan, and Germany. These collaborations increased the number of published papers and also improved the research quantities and qualities on TRPM7.

## Intellectual base

Citation networks have recently become a building block for a mathematical, graph-based theory of networks in the informatic sciences. In the bibliometric study, two components are contained in the conceptualized and visualized informatic sciences, including research fronts and intellectual bases [14].

Citations of medical journals by other journals are important for scientific publications. They are applied to calculate the impact factors of journals, which are viewed as a proxy for the scientific relevance and influence of an academic community [15]. From the TRPM7 paper with the most cited times over the past 15 years, they found that TRPM7 serves as a MLKL (Mixed Lineage Kinase Domain-like) downstream target to mediate Ca^2+^ influx and TNF-induced necroptosis, which reveals the critical mechanism of MLKL-mediated necroptosis [16]. The majority of other publications with high citation frequencies are overwhelmingly concentrating on the topic of ion channels, in particular for the flux of calcium and magnesium. A prospective study by SzydlowskaK from Toronto Western Hospital assessed that TRPM7 is an essential mediator of anoxic death in that blocking the Ca^2+^ permeable nonselective cation conductance (IOGD) or suppressing the TRPM7 expression is able to prevent the anoxic neuronal death [17].Jie Jin from Harvard University demonstrated that TRPM7 is the first TRP channel identified with a non-redundant role in embryogenesis and the only ion channel known to be necessary for thymopoiesis. The prototypical feature of TRPM7 is the permeabilization of Ca^2+^, Mg^2+^, and trace metals with a uniform structure that contains a kinase [18].

In the nervous system, TRPM7 contributes to a plethora of physiological and pathophysiological processes. In recent years, researchers have accumulated multiple evidences for an important role of TRPM7 in the mediation of neurotoxicity, neuro-injuries, and neuronal death. TRPM7 was reported to be involved in both excitotoxicity-related and neurodegenerative diseases such as epilepsy, Alzheimer’s disease (AD) and Parkinson disease (PD) [19,20]. In terms of neurological excitotoxicity, previous researches on neuronal anoxic, ischemia and traumatic brain injury demonstrated that inhibiting TRPM7 could promote the neuronal survival and decrease the neuronal death [21,22]. Considering the significant role of ions such as Ca^2+^, Mg^2+^ and Zn^2+^ in triggering epilepsy, the involvement of TRPM7 in epilepsy has also been discussed [23–25]. Researchers found that carvacrol, a natural inhibitor of TRPM7 channel, suppressed the recurrent epilepticus, early seizures, and hippocampal cell death, indicating the detrimental function of TRPM7 in mediating epilepsy. Neurodegeneration results were derived from a series of pathological events that lead to a progressive loss of neuronal structure and function associated with neuron injuries and death. Ca^2+^ overload-induced ROS production participating in cytoskeleton alterations and cell death has been shown to generate neurodegenerative disorders, including AD and PD. Overexpression of TRPM7 has been reported to increase H_2_O_2_-mediated injury, suggesting that TRPM7 is a potential target for neurodegenerative diseases [26].In addition, scarcity of Ca^2+^ and Mg^2+^is responsible for guamanian amyotrophic lateral sclerosis (ALS-G) and parkinsonism dementia (PD-G). In ALS-G and PD-G patients, researchers have disclosed a TRPM7 variant with the T1482I missense mutation. An increased sensitivity to inhibition by intracellular Mg^2+^ was observed in the heterologously expressed T1482I TRPM7 variant [27]. Notably, disfunction and loss of neuromuscular synapse have been illustrated to be one of the earliest pathological events in ALS [28]. TRPM7 is localized in the membrane of sympathetic neuronal acetylcholine (ACh)-secreting synaptic vesicles. As a part of the molecular complex of synaptic vesicle-specific proteins, the permeability of TRPM7 is also critical for sympathetic neurotransmitter release [29]. In summary, although the involvement of TRPM7 in the central nervous system (CNS) diseases has been characterized, specific mechanisms and clinical researches of CNS diseases are still not fully understood (Table 5).

**Table 5.** The mechanism of TRPM7 involved in central nervous system diseases.

Disease	Pathogenesis	The physiological processes/signaling pathways involved in TRPM7
Alzheimer disease(AD) [30–32]	Aβ deposition; Autophagy and metabolic dysfunction; Synapse damage and synaptic dysfunction; Imbalance of divalent cation in vivo; Oxidative stress; Tau protein hyperphosphorylation; Central cholinergic neuron injury and so on.	Improved the Mg^2+^ level, and adjusted the permeability of BBB-penetrating peptide and cause Aβ deposition decrease; Induced Ca^2+^ influx to enhance basal autophagy; Participated in the occurrence of oxidative stress.
Parkinson’s disease (PD) [20,33–35]	Substantia nigra dopamine neuron degeneration; Oxidative stress; Genetics;Environmentand so on	Activated pro-apoptotic signaling pathways and particaipated in neurodegeneration of dopaminergic neurons and terminals.
Amyotrophic Lateral Sclerosis (ALS) [29,36,37]	Motor neuron injury; Genetics;Environmentand so on.	Develop a molecular complex with vesicles and participate in regulating the release of neurotransmitters.
Stroke [38–41]	Glutamate receptor-mediated Ca^2+^ overload and neurotoxicity.	Key of neuronal injury in ischemic conditions: mediated Ca^2+^ toxicity, mediate Zn^2+^ toxicity.
Multiple sclerosis (MS) [42]	Inflammatory demyelination of white matter in the central nervous system.	Regulated the formation of glial scars and led to impaired nerve function.

TRPM7 has been abundantly expressed in tissues of cardiovascular system [21]. It is involved in cardiovascular functions and diseases such as cardiac fibrosis, vascular smooth muscle cell (VSMC) proliferation, Mg^2+^ homeostasis, and hypertension. It has been well established that Ca^2+^ is associated with fibrosis promotion. The enhanced expression of TRPM7 along with a striking potentiation in both TRPM7 currents and Ca^2+^ influx, is probably attributing to fibrogenesis and myofibroblast differentiation [43]. In addition, TRPM7 is responsible for ox-LDL-induced VSMC proliferation and migration through the phosphorylation of ERK1/2 and MEK1/2 [44]. Moreover, it has been suggested that TRPM7 plays a critical role in transcellular Mg^2+^transportation and Mg^2+^ homeostasis [45,46]. Reduction of Mg^2+^ contributes to the aldosterone-induced cardiovascular inflammation. An evidence suggested that TRPM7 could prevent cardiovascular fibrosis and inflammation induced by aldosterone via mediating Mg^2+^ transportation [46]. Furthermore, in primary human pulmonary artery smooth muscle cells, inhibition of TRPM7 contributes to the downregulation of MEK/ERK pathway, which causes proliferation promotion and apoptosis resistance, eventually provoking pulmonary arterial hypertension in hypoxia-induced rat model [47].

TRPM7 plays diverse oncogenic roles in a variety of cancers. In breast cancer, TRPM7 was reported to regulate the proliferation of tumor cells [48]. Moreover, the TRPM7 channel serves as a regulator of epithelial-mesenchymal transition (EMT) by meditating signal transducer, and as an activator of transcription 3 (STAT3) phosphorylation as well as vimentin expression. Inhibiting TRPM7 in breast cancer cell lines with 2-APB caused an increment in the percentage of cells in the S phase and a decreased cell population in the G0/G1 phase [49]. In prostate cancer, scientists reported that TRPM7 mediated-Mg^2+^ influx promoted the cell migration by inducing EMT, and TRPM7 suppression alleviated TGFβ-induced cell migration with a reduction of EMT markers [50]. Additionally, TRPM7 participates in pancreatic ductal adenocarcinoma (PDAC) cell invasion through regulating the Hsp90α/uPA/MMP-2 proteolytic axis [51]. In general, TRPM7 has a variety of functions in cancer cells including survival, cell cycle progression, proliferation, growth, migration, invasion, and EMT.

## Research frontiers

The frontiers of TRPM7 research were predicted using the strongest citation bursts of publications. CiteSpace V captured the keywords that were identified as research frontiers over time. The top four research frontiers of TRPM7 were ion channel, magnesium, secondary hypocalcemia (HSH), and proliferation.

The most striking feature of TRPM7 is the selectivity for divalent metal ions at hyperpolarized potentials. The majority of TRPM channels is permeable to Ca^2+^. The published paper showed that Ca^2+^ can enter the cells through the over-activation of glutamate receptors, a range of ion channels and transporters [17]. Ca^2+^ mediated by TRPM7 is associated with a variety of diseases such as heart disease and cancer. For example, the Ca^2+^ current induced by TRPM7 was often found to be potentiated in cancer cells, which is responsible for the development of Vacquinol-1 resistance in glioma cell lines. TRPM7 is also observed in the intracellular Zn^2+^ storage vesicles, and in this case, Zn^2+^ is the only permeable ion that is available to initiate inward currents through TRPM7 channels. TRPM7 can conduct several divalent cations, while its permeability to Zn^2+^ is higher than other ions [8]. The relationship between Mg^2+^ and the cell biological role of TRPM7 has been investigated. TRPM7-deficient cells tends to be Mg^2+^ deficient, and the reduced viability and proliferation of TRPM7-deficient cells can be rescued by supplementing extracellular Mg^2+^ [52]. TRPM7 is also related to Gd^3+^ which can inhibit TRPM7 channels.

Mg^2+^ homeostasis is essential for cell survival, and the loss of this regulation is linked to various neurodegenerative diseases [46]. TRP channels transmit extracellular signals into the cells. Recent publications indicated that extracellular Mg^2+^ enters endothelium mainly through the TRPM7 channel, TRPM6 channel, and MagT1 (Magnesium Transporter Protein 1) transporter [45,46]. Particularly in recent years, it has been shown that blocking TRPM7 channels seriously affected the Mg^2+^ homeostasis in cells [46].

Secondary hypocalcemia is a pediatric autosomal recessive disease that is always accompanied with hypomagnesemia and manifested as seizures and tetany [53]. Mutations in TRPM6 are the main cause of hypomagnesemia with secondary hypocalcemia [48]. Although there is no research data verifying that TRPM7 is directly related to secondary hypocalcemia, the proteins of TRPM6 and TRPM7 have a high sequence similarity and the TRPM6/TRPM7 hetero-oligomerization plays a role in TRPM6-mediated epithelial magnesium absorption [54]. Albeit that we found a pivotal evidence of TRPM7’s involvement in secondary hypocalcemia, researchers still need to further clarify the therapeutic approaches of secondary hypocalcemia.

The potential role of TRPM7 channels has been investigated in cell proliferation. TRPM7 has been shown to affect the proliferation through multiple ways in different kinds of cells such as lymphocyte, cancer cells and endothelial cells [55,56]. In the immune system, TRPM7 highly expresses in macrophages advances the production of interleukin-4 (IL-4) and macrophage colony-stimulating factor (M-CSF) to induce cell proliferation [57]. Apart from IL-4, TRPM7 also regulates other signals pertinent to inflammation, such as caspase-dependent cleavage and extracellular pH, but there are still no practical method to promote the proliferation of macrophages through regulating the function of TRPM7 [58].In previous studies, TRPM7 has also been proved to be able to affect the proliferation of cancer cells. Malignant tissues require elevated energy and Mg^2+^, accumulating Mg^2+^ at the expense of surrounding tissues, so Mg^2+^ intimately relate to the proliferation of cancer cells [59]. TRPM7 plays a critical role in Mg^2+^ regulation. With this regard, we can view TRPM7 as a key regulator of cancer growth, migration, and invasion. Despite these recent advancements, the expression of the TRPM7 channel and its role in proliferation remain poorly understood.

## Strengths and limitations

To the best of our knowledge, this study is the first bibliometric analysis focusing on TRPM7 trends. There has been no time limitation for our literature retrieval, the data downloaded from WoS covered the vast majority of articles in the field of TRPM7 research, and the data analysis was relatively objective and comprehensive, clearly showing the status of TRPM7. However, this study exclusively consisted of original articles and reviews published in 2004–2019 and was indexed by WoS database. Since books, conference abstracts, and other types of publications have not been included in the range of document screening, our data may not represent all literatures. Furthermore, we only included articles written in English in our analysis, making the analysis incomplete in the worldwide perspective. In terms of retrieval of databases, we only retrieved publications from the WoS database. Although other databases such as PubMed, Scopus and Embase could provide a broader range of coverage of scientific literatures, WoS CC is superior to providing detailed data (*e.g*., annual publications, author information, journal sources, country, and institution information). Importantly, the results of this study were stable and highly reproducible. Now that this study covers the vast majority of papers from 2004 to 2019, the latest publications may not affect the final results.

We carried out a series of scientific maps of journals, countries/regions, institutions, authors, co-cited references, and citation burst keywords to identify the theme evolution and trends in the intellectual landscape of this domain. TRPM7 is a subject that deserves a thorough development due to its promising and brilliant future. A considerable number of papers have been published in highly influential journals. The USA exerted an important influence on this domain. The University of Toronto, University of Hawaii and Harvard University has been identified as excellent institutions for research collaborations. Gudermann T and Chubanov V are prominent scientific leaders in this research field. Organizations can refer to this article as a reference when deciding whether or not to provide repeatable supporting funding to a given research team. Additionally, institutions should integrate and complement their research fields on TRPM7 channels.

## Geolocation information

Shenyang, Liaoning, China
